# A potential therapeutic peptide-based neutralizer that potently inhibits Shiga toxin 2 *in vitro* and *in vivo*

**DOI:** 10.1038/srep21837

**Published:** 2016-02-23

**Authors:** Tao Li, Wei Tu, Yuenan Liu, Peng Zhou, Kun Cai, Zhan Li, Xiong Liu, Nianzhi Ning, Jie Huang, Shenghan Wang, Jian Huang, Hui Wang

**Affiliations:** 1 State Key Laboratory of Pathogens and Biosecurity, Beijing Institute of Microbiology and Epidemiology, Fengtai District, Beijing, PR China; 2 Guangxi Medical University, Nanning, PR China; 3 Department of Microbiology, Anhui Medical University, Hefei, 230032, PR China; 4 Center of Bioinformatics (COBI), Key Laboratory for NeuroInformation of Ministry of Education, University of Electronic Science and Technology of China, Chengdu 610054, China; 5 Center for Information in Biomedicine, University of Electronic Science and Technology of China, Chengdu 610054, China; 6 Department of Urology, Beijing Xuanwu Hospital, Beijing, People’s Republic of China

## Abstract

Shiga toxin 2 (Stx2) is a major virulence factor in infections with Stx-producing *Escherichia coli* (STEC), which can cause serious clinical complications in humans, such as hemolytic uremic syndrome (HUS). Recently, we screened and identified two peptide-based Stx2 neutralizers, TF-1 and WA-8, which specifically and directly bind to Stx2. Computer simulations suggested that the majority of TF-1 or WA-8 binds tightly at the receptor-binding site 3 of Stx2. The two peptides also effectively inhibited the cytotoxic activity of Stx2 by blocking the binding of Stx2 to target cells. TF-1 exhibits remarkable therapeutic potency in both mice and rat toxicity models. In mice toxicity models, TF-1 provided full protection when mice were injected with 5 LD_50_ of Stx2. In rat toxicity models, TF-1 reduced fatal tissue damage and completely protected rats from the lethal challenges of Stx2. In these rats, TF-1 significantly decreased the concentration of Stx2 in blood and diminished tissue distribution levels of Stx2. Furthermore, TF-1 effectively protected rats from the pathological effects caused by Stx2, especially in the kidney, thymus, adrenal gland, and lung. Taken together, these results indicate that TF-1 is a promising therapeutic agent against the pathogenicity of Stx2.

Shiga toxin (Stx) is a major virulence factor of Stx-producing Escherichia coli (STEC)^1^, including Enterohaemorrhagic *E. coli* (EHEC). A large outbreak of STEC-HUS caused by *E. coli* O104: H4 have occurred in Germany in May 2011^2^,^3^. Stx has been shown to be responsible for watery or bloody diarrhea, hemorrhagic colitis, and hemolytic uremic syndrome (HUS)^4^,^5^.

Two major classes of Shiga toxins (Stx1 and Stx2) have been identified in EHEC. Both types of toxins have similar structures and modes of action. Shiga toxins possess an AB5 structure, composed of a pentamer of subunit B linked to a single A subunit. The A subunit is responsible for the enzymatic activity of the toxin: inhibition of protein synthesis. Whereas the B-pentamer plays a vital role in binding to the functional cell surface receptor globotriaosylceramide Gb3^6^. The 3D structure of Stx reveals the presence of three distinctive binding sites (sites 1, 2, and 3) on each B subunit monomer for the trisaccharide moiety of Gb3^7^. On one hand, Shiga toxin (Stx) trigger signaling events that lead to apoptosis or programmed cell death in intestinal cells^8^. On the other hand, the intestinal epithelial injury was caused by Stx, Bacterial lipopolysaccharide (LPS) or inflammatory cytokines may promote Stx passage across the intestinal epithelial cells into the blood circulation, and then causes gastrointestinal, central nervous systems and higher levels of Gb3 receptor organs such as kidney and brain damage^9^. Epidemiological and experimental studies have suggested that Stx2 is more clinically significant and more toxic than Stx1. Stx2-producing strains are more frequently associated with the development of HUS than are Stx1-producing strains^10^.

The current treatment for EHEC infection, antibiotics, is not optimal considering that antibiotics may not change the course of the infection and may even increase the incidence of HUS caused by the pathogen. This untoward effect has been proposed to be mediated by antibiotic-induced bacteriolysis and the release of intracellular Shiga toxins. Therefore, the US CDC recommends using non-antibiotic treatments against an EHEC infection. However, there is no effective treatment until now, and only supportive management is used clinically^11^,^12^,^13^. Recently, several therapeutic options such as an active immunization with Stx toxoids^14^,^15^,^16^, a passive immunization with Stx antibodies^17^,^18^,^19^, and Gb3 receptor analogues^20^,^21^,^22^ have been developed. Unfortunately, while these approaches have shown to be effective in animal testing, clinical trials are lacking. Another possible therapy to treat EHEC infection in humans would be the development of a novel, effective Stx neutralizer, which specifically binds to and clears Stx from the circulation.

As a drug, peptide compounds are gaining more and more attention due to the technical advantages such as low molecular weight, inexpensive mass production costs using chemical synthesis or genetic engineering methods, low immunogenicity, mild side-effects, the diversity of administration routes, and easy absorption. Some peptides derived from library screenings often modulate the target’s activity *in vitro* or *in vivo* and can be used in drug design and as alternatives to antibodies^23^,^24^,^25^,^26^,^27^,^28^. Therefore, we sought to find a peptide-based agent to target Stx2B in this study. First, various, novel peptides binding to recombinant histidine-tagged Stx2B subunit (2BH) were selected from a phage display library, after which all peptides were examined with a subtraction procedure screening. We synthesized several peptides which bound to 2BH and investigated their interactions with Stxs using an enzyme-linked immunosorbent assay (ELISA), 3-(4, 5-Dimethylthiazol-2-yl)-2, 5-Diphenyltetrazolium Bromide (MTT) assay, and computer simulations. We have found that the peptide TFNMWLPTFNQW (TF-1, Patent No. US8946384B2, AU2011304941B2 and CN102040654B) binds Stx-2 strongly and neutralizes Stx-2 effectively. The biological activity of TF-1 was then evaluated in animal testing. TF-1 protected rats from lethal challenge with a fatal dose of Stx2 and effectively neutralized Stx in the circulation, suggesting that TF-1 is a promising therapeutic agent against infections by Stx.

## Materials and Methods

### Materials

Recombinant Stx2 and Stx2B subunit (2BH) were produced as described previously^16^,^29^. FITC-2BH was prepared by labeling 2BH with fluorescein isothiocyanate (FITC). Specifically, the purified 2BH mentioned above was dialyzed in a sodium carbonate buffer (pH 9.2) after which FITC in DMSO was added (0.05 mg FITC/ 1 mg protein). The mixture was stirred for 2 h in the dark at RT and then dialyzed in a phosphate buffer (pH 7.2) during which the buffer was repeatedly renewed until no FITC diffused through the membrane at 4 °C. I-labeled Stx2 (^125^I-Stx2) (Amersham Co., Sweden) was prepared as described previously. Mouse anti-Stx2B polyclonal serum was prepared in our lab.

### Ethics statement

The Beijing Institute of Microbiology and Epidemiology Animal Care and Use Committee approved our study protocol. The animal care and use protocol adhered to the regulations of the Institutional Animal Care and Use Committee (IACUC). BALB/c mice (weighing 17–19 g) and Wistar rat (weighing 175–185 g) were purchased from the Laboratory Animal Center of the Academy of Military Medical Sciences, Beijing, China. The animals were fed with standard diet and water, maintained under the following conditions: 12 h light/12 h dark controlled lighting, 24 °C to 28 °C temperature, and 55% relative humidity. All animals were handled under the care and supervision of a veterinarian. The mice or rat that were severely injured by high dose of toxin injected, were sacrificed by cervical dislocation at the end of experiment.

### Screening of peptides for Stx2B subunit (2BH)

Biopanning was carried out according to the procedures of a phage display library kit (New England Biolabs Inc., Cambridge, MA), which is based on a combinatorial library of random peptide 12-mers fused to a minor coat protein (pIII) of the filamentous coliphage M13. The phage library (5.0 × 30 × 10^10^ transducing units (TU)) was added to the S-adenosyl-L-methionine (SAM) substrate. After incubation for 1 h at RT, the SAM substrate was washed three times with Tris-buffered saline containing 0.1% Tween 20 (TBS-T). The phages bound to Stx2B were eluted with 0.1 N Glycine-HCl buffer (pH 2.2). The eluate was neutralized with 1 M Tris–HCl buffer. The phage clones obtained were amplified through infection into host bacterial cells (*E. coli* ER2738). Thus, five rounds of screening were performed to enrich the targets of Stx2B. Peptide-displaying phage clones were tested by ELISA. The clones binding to Stx2B with relative higher affinity were selected from the fifth round of screening and then sequenced to identify their amino acid sequences.

### Peptide synthesis

All peptides (Table 1) were synthesized by a solid-phase method using standard Fmoc chemistry on Wang resin. Coupling of amino acids was carried out using 1-hydroxybenzotriazole hydrate (HOBt) and benzotriazole-1-yloxy-tris (dimethylamino) phosphonium hexa-fluorophosphate (BOP) in DMF. The Fmoc group was removed in 30% piperidine/DMF. Peptides were purified by reversed-phase HPLC with a gradient of acetonitrile/0.1% TFA. The identity and purity of all peptides were confirmed by electrospray ionization mass spectrometry and reversed-phase high-performance liquid chromatography.

### ELISA analysis of the binding between 2BH and inhibitory peptides

The indicated concentration of inhibitory peptides (TF-1 and WA-8), dissolved in PBS, was coated onto each well of a 96-well ELISA plate (for 24 h at 4 °C). After blocking, the plate was incubated with Stx2B (0.1 μg/ml) for 1 h at RT. Bound Stx2B was detected using mouse anti-Stx2B polyclonal serum. Five Stx2 mutants with mutations at Asp16 (site 1), Asp17 (site 3), Trp33 (site 3), Asn34 (site 3), Gly61 (site 2) have been conducted and expression. The binding of TF-1 or WA-8 with Stx2B or Stx2B mutant were examined using ELISA.

### Neutralization of Stx2 in HeLa cells with inhibitory peptides

After HeLa cell adhesion on 96-well microplates, the medium was removed and replaced with a mixture containing DMEM (100 μl/well), a range of inhibitory peptide (TF-1 and WA-8) concentrations (4.68–300 μM) and 100 μl of 25 ng/ml (about 5 CD_50_) Stx2. The plates were incubated under 5% CO_2_ at 37 °C for 2 days. The supernatant was removed, and fresh DMEM (containing 0.5 mg/mL MTT) was added (20 mL/well) and incubated at 37 °C for 4 h. The supernatant was removed and DMSO was added (150 mL/well). After incubation for 10 min, the absorbance was read at 570 nm. Cell viabilities were calculated according to the formula: Inhibition rate % = [(OD_peptide + Stx2_−OD_Stx2_)/(OD _control_)−OD_Stx2_].

### I-Stx2 binding assay

HeLa cells grown in a 24-well plastic microplate were treated with ^125^I-Stx2 (5 × 10^5^ cpm/μg of protein) in the absence or presence of a desired amount of TF-1 or WA-8 for 30 min at 4 °C. After extensive washing, cells were dissolved in lysis solution (0.1 M NaOH and 0.5% SDS), and the recovered radioactivity was measured by a gamma counter.

### Intraperitoneal administration of Stx2 to BALB/c mice

Four-week-old female BALB/c mice weighing 17–19 g (obtained from the Animal Center of the Institute of Microbiology and Epidemiology, Academy of Military Medical Sciences, Beijing, China) were randomly separated into six groups. TF-1 or WA-8 efficacy was evaluated by administering either at a dose range of 0.7 mg/kg–22 mg/kg of body weight in 200 μl of PBS or 200 μl of PBS alone (control) intraperitoneally to each mouse followed by intraperitoneal administration of 5 LD_50_ or 10 LD_50_ (30 or 60 ng) of Stx2 20min later. The data were analyzed by Kaplan–Meier survival analysis, or if no mice had died by the end of the observation, by Fisher’s exact test. The animal experiments reported here and mentioned below were conducted according to the principles set forth in the Guide for the Care and Use of Laboratory Animals^30^.

### Intravenous administration of Stx2 to Wistar rats

A lethal dose of Stx2 (≥1LD_100,_ 100ng) was administered to 14–30 male Wistar rats (175–185 g, obtained from the Animal Center of the Institute of Microbiology and Epidemiology, Academy of Military Medical Sciences, Beijing, China) through a tail vein with or without the desired amount of TF-1, and the survival rates of the mice were monitored. The data were analyzed and the remaining steps were performed as described in above paragraph. The kidneys and other tissues were removed, fixed in neutral-buffered formalin, then were paraffin-embedded, and sectioned. All isolated tissues were stained with hematoxylin and eosin (H&E).

### Effect of TF-1 on serum clearance and tissue distribution of ^125^I - Stx2

A group of Wistar rats (n = 3) were injected intravenously with either a mixture of TF-1 (2 mg/rat) and ^**125**^I-Stx2 (≥1LD_100_, 5 × 10^5^ cpm/μg of protein) in 200 μl saline or 200 μl of ^**125**^I-Stx2 (≥1LD_100_) in saline alone (control). In these studies, all of the injected doses markedly surpassed the 1LD_100_ value. Pilot experiments were performed to examine toxin distribution at 0.5 or 72 hours post injection and the effects of perfusion prior to tissue collection on the biodistribution data were obtained from the treatment group (TF-1 + Stx2) and attacked group (Stx2). 300 μl blood samples were subsequently obtained from the lateral saphenous vein at regular intervals covering a time scale between 5 min and 72 h (sampling: 5 min, 10 min, 15 min, 30 min, 1 h, 2 h, 4 h, 8 h, 12 h, 24 h, 48 h, and 72 h). Upon taking the last blood sample, rats were sacrificed immediately by cervical dislocation. Tissues/organs (including the thyroid gland, thymus, heart, lung, liver, spleen, adrenal gland, kidney, bladder, testis, lymph node, intestine content, jejunum, fat, muscle, spinal cord, cerebellum, nasal turbinates, and eyes) of these animals were subsequently removed and weighed, following which the amount of radioactivity (cpm/g) present in these tissues/organs was determined using a gamma counter. All blood samples were centrifuged immediately upon collection. To monitor for signs of renal dysfunction and hepatic injury, plasma samples were analyzed for blood urea nitrogen (BUN), creatinine (CRE), and alanine aminotransferase (ALT). The biochemical tests were done at the clinical laboratory, 307 Hospital of PLA (Beijing, China).

TCA precipitation: The percent of degradation of circulating ^125^I- Stx2 was calculated based on the change in trichloroacetic acid (TCA) precipitability of plasma radioactivity over time as previously described. Chilled saline (200 ml) and chilled TCA solution (200 ml; 40%, w/v) were added sequentially to a plasma sample. The mixture was placed on ice for 30 min, and then centrifuged at 3000 rpm for 20 min. The amount of radioactivity of intact Stx2 in the pellets was assessed using a gamma counter, as was the amount of radioactivity in the supernatants (considered to be degraded Stx2). The percentage of radioactivity precipitated by TCA was then calculated using the formula: precipitated radioactivity/total radioactivity ×100%.

### Computer simulation

The atomic-level structures of peptides TF-1/WA-8 were predicted using the *de novo* peptide folding server PEP-FOLD (http://bioserv.rpbs.univ-paris-diderot.fr/services/PEP-FOLD/)^31^. The protein–protein docking method ZDOCK (http://zdock.umassmed.edu/) was employed to perform a coarse-grained search for the interaction modes of Stx2B with peptides TF-1/WA-8^32^. The docked complex structure models of Stx2B with peptide ligands TF-1/WA-8 were further refined in a computationally expensive manner with the FlexPepDock server (http://flexpepdock.furmanlab.cs.huji.ac.il/)^33^, which combined Monte Carlo sampling and the Rosetta force field to minimize the structure of the protein–peptide systems to achieve their global energy minimum, resulting in a conformation assembly for the systems. The complex systems of Stx2B with peptide ligands TF-1/WA-8 were subjected to atomistic molecular dynamics (MD) simulations in the framework of AMBER09 biomolecular force field. The binding free energy, ΔG, of peptide ligands TF-1/WA-8 to Stx2B was derived from the MD snapshots of complex dynamics trajectories using the MM/GBSA method. The non-bonded interactions between Stx2B and TF-1/WA-8 were computed with the molecular mechanism (MM) approach, which included the van der Waals potential and electrostatic energy, while the desolvation effect due to the Stx2B–TF-1/WA-8 binding was described by the generalization Born (GB) model (for polar contribution) and the surface area (SA) strategy (for nonpolar contribution).

## Results

### Peptides TF-1 and WA-8 specifically and directly bind to the Stx2B subunit (2BH)

**Figure 1:**
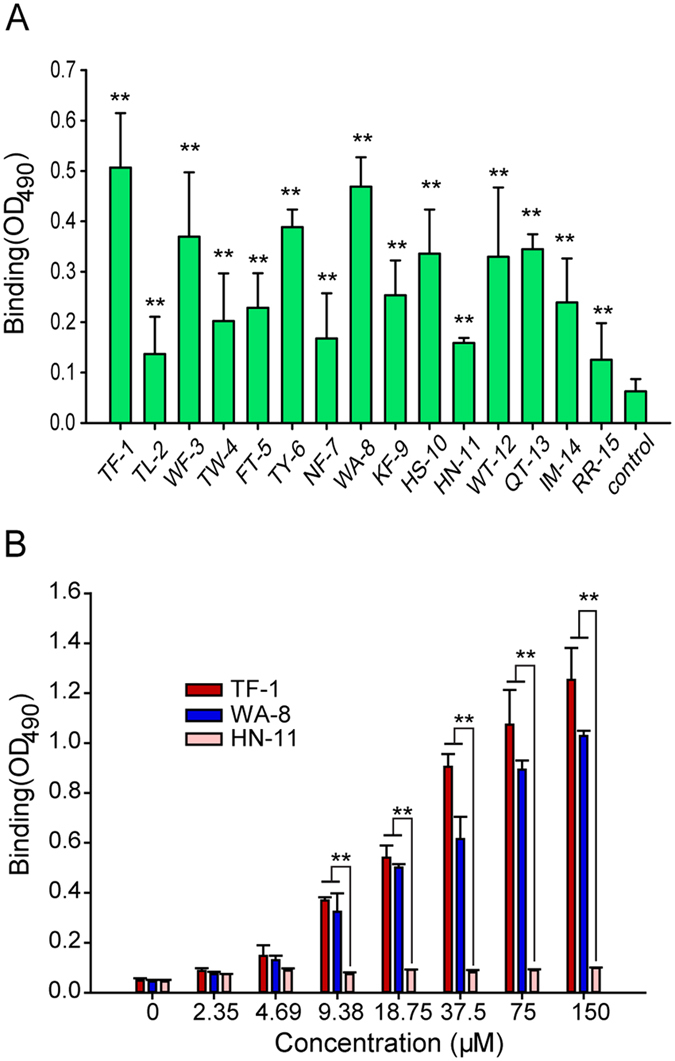
Peptides TF-1 and WA-8 specifically and directly bind to 2BH. (**A**) Initially, all peptides binding to 2BH were screened by ELISA. The binding (OD490) of different peptides was compared with control. **p < 0.01. (**B**) Binding of the TF-1or WA-8 with different concentrations to 2BH was examined by ELISA (mean ± SEM, n = 3). Irrelevant peptide HN-11 was used as a control. *p < 0.05, **p < 0.01.

In this study, several novel peptides binding to recombinant histidine-tagged Stx2B subunits (2BH) were selected from a phage library (Table 1). Mass spectrometry and high performance liquid chromatography were used as quality control tools for the synthesized peptides (Fig. S1). The activity of each peptide binding to 2BH was preliminarily screened by ELISA (Fig. 1A).

We found that two peptides, TF-1 and WA-8, had potent Stx2B-binding activity. Thus, we pursued these two candidates to assess their potential as potent inhibitors. As shown in Fig. 1B, peptides TF-1 and WA-8, respectively bound to 2BH in a dose-dependent manner. However, TF-1 binding to 2BH was stronger than WA-8 in a range of concentrations (37.5–150 μM), while the binding of the control, HN-11, to 2BH did not significantly change in a range of concentrations (2.35–150 μM). Taken together, we assume that TF-1 and WA-8 specifically recognized Stx2B subunit (2BH).

### Peptides TF-1 and WA-8 effectively inhibit the cytotoxic activity of Stx2 by blocking the binding of Stx2 to target cells

**Figure 2:**
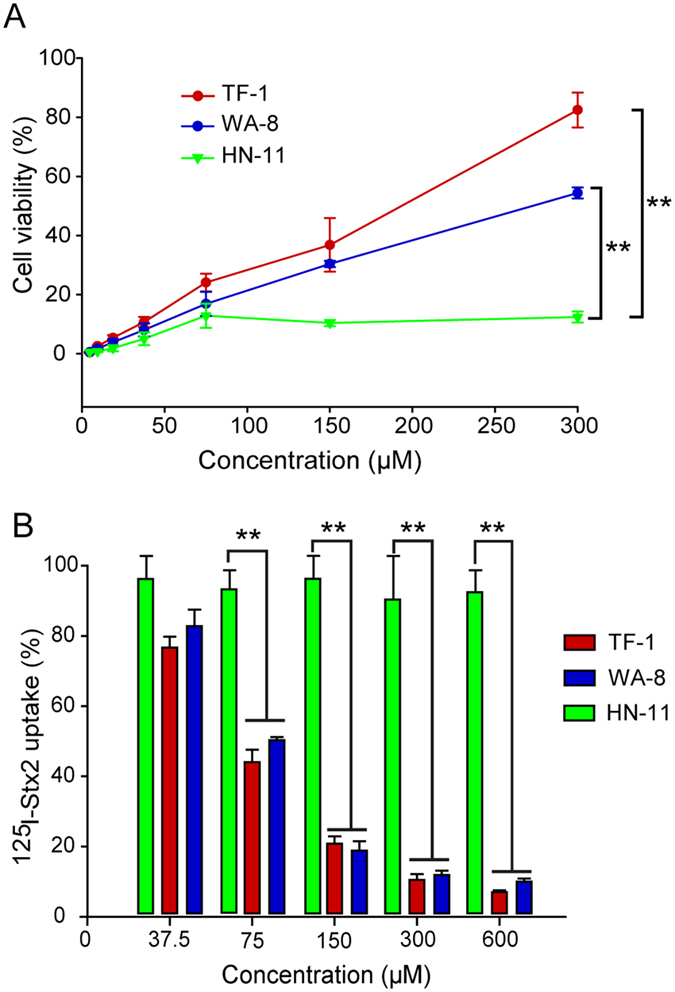
Peptides TF-1 and WA-8 effectively inhibit the cytotoxic activity of Stx2 by blocking the binding of Stx2 to target cells. (**A**) TF-1 and WA-8 inhibited Stx2 cytotoxicity in HeLa cells. Survival rates of HeLa cells against Stx2 were determined in the presence of various concentrations of TF-1 or WA-8. Irrelevant peptide HN-11 was used as a negative control. **p < 0.01. Data are presented as a percentage of the control value (mean ± SEM, n = 3). (B) Inhibitory effect of TF-1 or WA-8 on the uptake of ^125^I-Stx2 in HeLa cells. HeLa cells were incubated with ^125^I-Stx2 (3 × 10^5^ cpm/μg, 0.5 μg/ml) and different concentrations of TF-1 or WA-8. The inhibitory effect of TF-1 or WA-8 was compared with the control (peptide HN-11). **p < 0.01. The data are presented as mean ± SEM (n = 3).

TF-1 and WA-8 effectively inhibited the cytotoxic activity of Stx2 toward HeLa cells, one of the cell types most sensitive to Stx2. As shown in Fig. 2A, HeLa cells were incubated with a mixture of 5 × CD_50_ Stx2 (25ng/ml) as well as TF-1 or WA-8 in a range of concentrations (4.68–300 μM) for 48 h. The percentage of cell survival against Stx2 was increased by the addition of TF-1 or WA-8. The addition of the maximum concentration of 300 μM TF-1 and WA-8 conferred 82% and 54% protection of HeLa cells in the presence of Stx2, respectively. Hence, the ability of TF-1 and WA-8 to protect HeLa cells against the infection with Stx2 was speculated to be due to the neutralizing effect of the peptides. TF-1 demonstrated a stronger protective effect than WA-8. The IC_50_ values of TF-1 and WA-8 were 180 μM and 273 μM, respectively. Almost no inhibitory effect was observed with peptide HN-11 in a range of concentrations (4.68–300 μM).

Next, a ^125^I-Stx2 binding assay was used to detect the effect of peptides on the uptake of Stx2 in cells. TF-1 or WA-8 markedly inhibited the binding of Stx2 (^125^I-Stx2) to HeLa cells in a dose-dependent manner. With 300 μM TF-1 or WA-8, ^125^I-Stx2 binding to the cells was almost completely abrogated (>90%, Fig. 2B). Taken together, these results indicate that TF-1 or WA-8 effectively inhibit the cytotoxic activity of Stx2 by blocking the binding of Stx2 to target cells. We also observed almost no inhibitory effect on the uptake of Stx2 with the peptide HN-11 in a range of concentrations (37.5–600 μM).

### Peptide TF-1 effectively protected mice and rats from the lethality caused by Stx2

**Figure 3:**
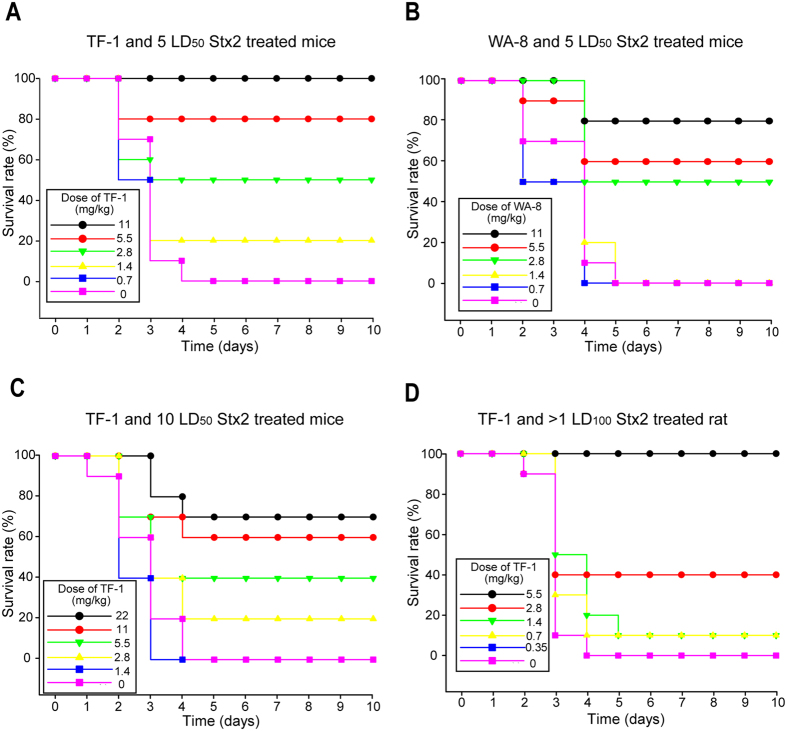
Protective effect of TF-1 or WA-8 on the survival of Stx2-treated mice or rats. (**A**) Dose-dependent efficacy of TF-1 when administered 20 min before mice were injected with 5 LD_50_ of Stx2 (n = 10/group). (**B**) Dose-dependent efficacy of WA-8 when administered 20 min before mice were injected with 5 LD_50_ of Stx2 (n = 10/group). (**C**) Dose-dependent efficacy of TF-1 when administered 20 min before mice were injected with 10 LD_50_ of Stx2 (n = 10/group). (**D**) Dose-dependent efficacy of TF-1 in rats administered a mixture of TF-1 and Stx2 (≥1 LD_100_) (n = 10/group).

Next, the inhibitory effects of TF-1 and WA-8 on the lethality of intravenously administered Stx2 to mice and rats were investigated. First, we carried out intraperitoneal administration of Stx2 to BALB/c mice. Dose-dependent efficacy of TF-1 or WA-8 when administered 20 min before mice were intraperitoneal administered 5 LD_50_ of Stx2 was investigated. TF-1 can provide full protection at the dose of 11 mg/kg (Fig. 3A). Compared with TF-1, WA-8 provided weaker protection at the same dose (protected 80% of the mice, Fig. 3B), which is in concordance with the results of *in vitro* studies. Without treatment, 100% of the challenged mice died within 5 days (survival time of mice without treatment with TF-1 or WA-8: 3.8 ± 0.2 days (mean ± SEM), *p* < 0.001, Fig. 3A,B). Then, the protection activity of TF-1 was detected in mice injected with 10 LD_50_ of Stx2. At this dose of Stx2, TF-1 only could provide partial effective protection. There was a 70% survival rate among mice at the dose of 22 mg/kg of TF-1 (Fig. 3C). In marked contrast, 100% of the challenge mice died within 5 days without treatment (average survival period of mice not given TF-1: 3.7 ± 0.3 days, *p* < 0.0001, Fig. 3C).

To further evaluate the potential *in vivo* efficacy of TF-1, TF-1 was used to inhibit Stx2-induced mortality in rats. A mixture of TF-1 and Stx2 (≥1 LD_100_) was intravenously injected into rats. Dose-dependent efficacy of TF-1 was observed in Stx2-treated rats. Moreover, TF-1 could provide full protection at a dose of 5.5 mg/kg. In stark contrast, 100% of the challenge rats died within 4 days (average survival time of mice not given TF-1: 3.0 ± 0.2 days, *p* < 0.0001, Fig. 3D), while the TF-1 treated rats survived more than 20 days without any pathological symptoms (data not shown). TF-1 itself did not affect the viability of the control group (data not shown).

### Peptide TF-1 effectively protected rats from the pathology caused by Stx2

Stx2 causes multifocal vascular damage in different tissues, which is closely related to its morbidity and mortality in animal models^1^,^4^,^22^. Therefore, morphological, anatomical, and pathological changes in different tissues were investigated after the intravenous administration of Stx2 into rats with or without TF-1. Morphological and anatomical studies showed that intravenous injection of Stx2 induced diarrhea 1–2 days post-injection (Fig. S2–A2) and resulted in death 3–4 days post-injection. In these rats, inflammation was observed in the cecum, and the stomach exhibited gastric retentions (Fig. S2–B2). The kidney was heavily swollen with reduced elasticity and luster (Fig. S2–C2). Co-administration of TF-1 with Stx2 completely protected rats from this pathology (Figs. S2–A3, B3, C3). The stomachs of rats treated with TF-1 had only slight gastric retention and cecum from rats treated with TF-1 showed normal structure when compared to control rats. Thus, the gastric retention induced by Stx2 was reversed by TF-1. Moreover, Kidneys from rats treated with TF-1 showed normal structure when compared to control rats. These pathological changes are in line with the histopathology results.

**Figure 4:**
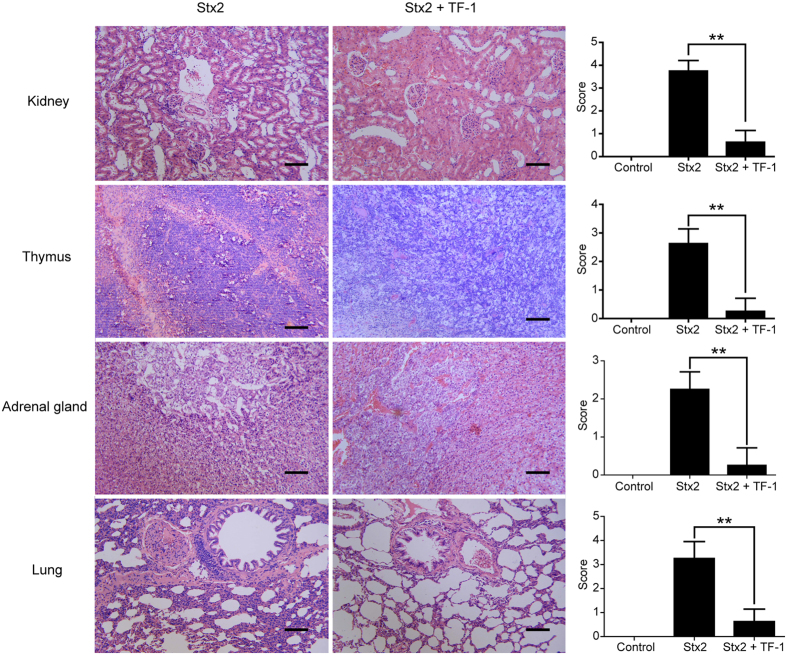
Histopathological studies in the kidney, thymus, adrenal gland, and lung of rats with or without TF-1 treatment. The pathology of with different tissues has been blindly scored by a veterinary pathologist. A scale from 0 to 4 was used with 0 being the least severe or lowest, and 4 being the most severe or highest (n = 8/group). The blind score of stx2 and TF-1 treated tissues was compare with only stx2 treated ones. **p < 0.01.

Histopathological examination of the kidney tissue obtained from rats treated with Stx2 indicated the development of kidney lesions characteristic of Stx toxicosis and the development of tubular necrosis by 72 h as previously reported^31^. After administration of Stx2, the kidneys of the rats showed congestion in glomeruli, interstitial hemorrhage, extensive cell fibrosis, and epithelial necrosis in collecting ducts (Fig. 4). Co-administration of TF-1 with Stx2 completely protected rats from this pathology. Furthermore, the congestion and interstitial hemorrhage disappeared after TF-1 treatment and the fibrous and necrotic cellular phenotypes in the kidneys were also reversed (Fig. 4). However, denaturalization and depauperation were sometimes observed in the epithelial cells of many tubules (data not shown).

Moreover, histopathological examinations of tissue samples from the thymus revealed serious thymic lesions in all rats treated with Stx2, which is in line with previous findings from our group^31^. In all Stx2-treated rats, the thymus shrank considerably, and the medulla and cortex were hard to differentiate. The thymus from the treatment group co-administered the neutralizing peptide TF-1 with Stx2 recovered to normal (Fig. 4). Despite the congestion, interstitial hemorrhages disappeared after TF-1 treatment, and the fibrous cells and necrotic cells in the thymus were also reversed, while there was still denaturalization and depauperation sometimes observed in the epithelial cells of many tubules. It was unexpected to us that TF-1 would provide complete protection of the thymus demonstrated in macroscopic pathological and in histopathological examinations (Fig. 4). A similar histopathological result was found on adrenal gland and lung tissue (Fig. 4).

In addition, histopathological examinations were performed on many other tissues of treated rats (S3 Fig.). Besides the kidney, thymus, adrenal gland, and lung, there were no obvious pathological changes in other tissues of Stx2-treated rats such as cerebellum, cerebrum, liver, spleen, heart, intestine, stomach, fat, spinal cord, and testis.

### TF-1 significantly decreases the concentration of Stx2 in plasma and tissue of treated rats

**Figure 5:**
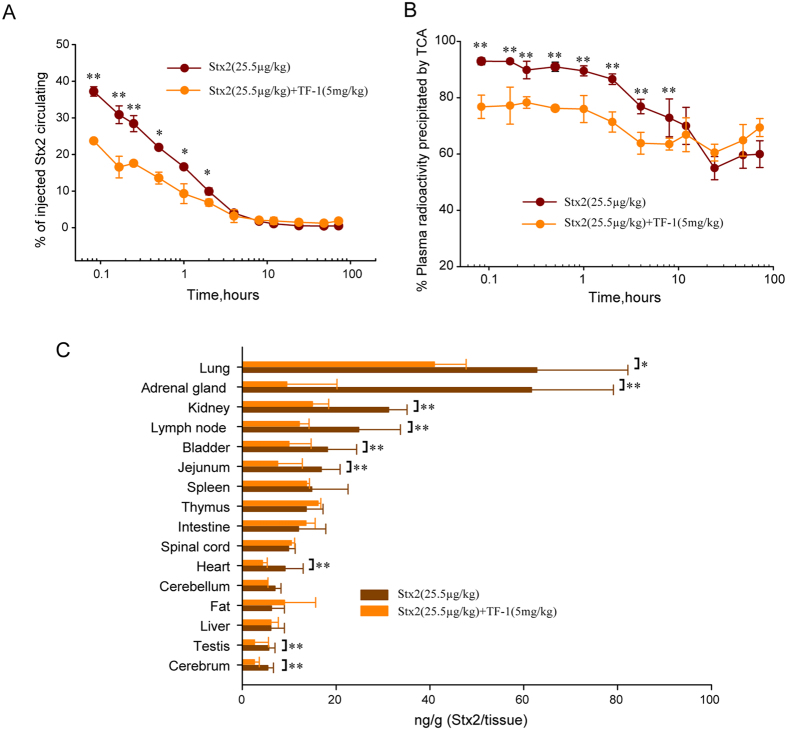
Effect of TF-1 on clearance of ^125^I-Stx2 in rats. (**A**) Effect of TF-1 on serum clearance of ^125^I-Stx2. A mixture of 5 mg/kg TF-1 and 25.5 μg/kg ^125^I-Stx2 were injected into rats intravenously (n = 3), and the counts remaining in circulation were compared with those from rats (n = 3) that were challenged with ^125^I-Stx2 alone over 72 h. *p < 0.05, **p < 0.01. (**B**) Degradation of circulating ^125^I-Stx2 after administration of ^125^I-Stx2 and a mixture of TF-1 and Stx2. Degradation of circulating ^125^I-Stx2 was monitored by the change in TCA precipitability of plasma radioactivity over time. Each data point represents the mean ± SD. Degradation of circulating ^125^I -Stx2 in the rats injected with 5 mg/kg TF-1 plus 25.5 μg/kg Stx2 (n = 3) was compared to those treated with 25.5 μg/kg Stx2 alone. *p < 0.05, **p < 0.01. (**C**) Effect of TF-1 on tissue distribution of ^125^I-Stx2. 5 mg/kg TF-1 mixed with 25.5 μg/kg ^125^I-Stx2 were injected into rats (n = 3). After 72 hours, the toxin distribution was compared with the challenged group that was injected with 25.5 μg/kg ^125^I-Stx2 alone. *p < 0.05, **p < 0.01.

To examine the effect of TF-1 on the plasma and tissue distribution of Stx2, we injected ^125^I-Stx2 intravenously into rats with or without TF-1. The percentage of injected toxin circulating in the treatment group (5 mg/kg TF-1 plus 25.5 μg/kg ^125^I -Stx2) was significantly lower than in the Stx2 challenged group (only 25.5 μg/kg ^125^I -Stx2) over the first 4 hours (Fig. 5A, *p* < 0.01 or *p* < 0.05). These results suggest that TF-1 could shorten the half-life and reduce the amount of Stx2 *in vivo*. The analysis with DAS 2.0 software showed that the treatment group belonged to a two-compartmental model (Table 2). Both the elimination half-life (1.684 h of TF-1 vs. 27.876 h of Stx2, *p* < 0.01) and AUC (area under concentration-time curve, 243.545 mg/l·h of TF-1 vs. 846.395 mg/l h of Stx2, *p* < 0.01) of the treatment group were shorter or smaller than that of Stx2 challenged group (Table 2). These results also indicate that TF-1 could shorten the half-life and reduce the amount of Stx2 *in vivo*. Moreover, the lower percentage of TCA-precipitable radioactivity of the intervention group over the first 8 hours (Fig. 5B, *p* < 0.01) suggests that TF-1 has a possible effect on accelerating the metabolism of Stx2 *in vivo*.

Furthermore, the accumulation of radioactivity in different tissues of rats 72 h after Stx2 treatment was detected. The concentration of Stx2 in the kidney and cerebrum, well-known Stx primary target organs, was reduced by 2.12 and 2.09 times, respectively (Fig. 5C, *p* < 0.05). Simultaneously, we also observed that the treatment group showed significantly lower concentrations of Stx2 in the lung, adrenal gland, kidney, lymph node, bladder, and jejunum compared to the challenged group (Fig. 5C, *p* < 0.01).

### The computer simulated interaction between Stx2B and peptides TF-1 or WA-8

**Figure 6:**
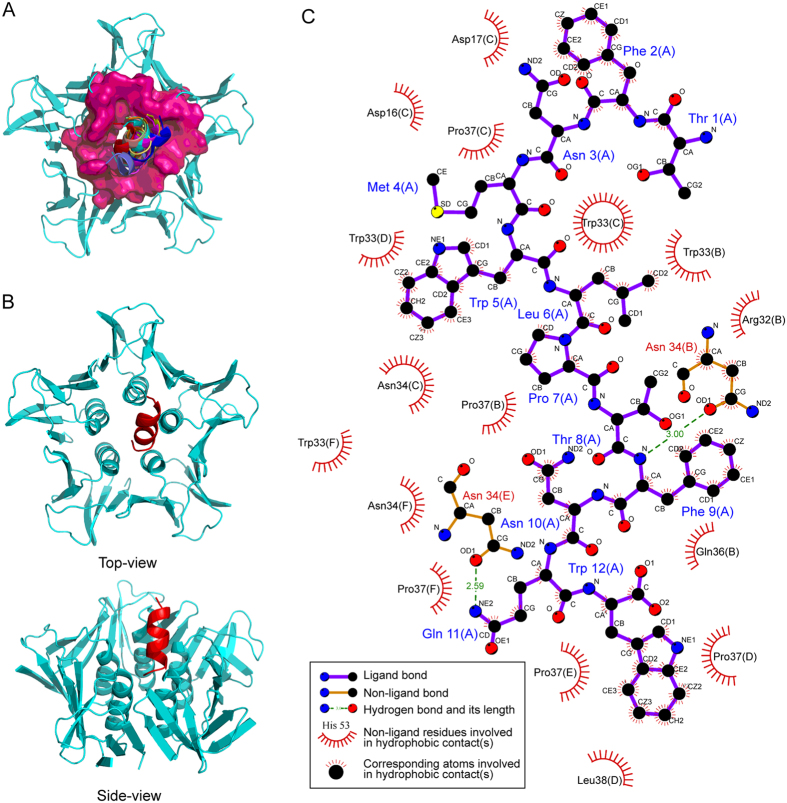
Computer-simulated binding mode between TF-1 and Stx2B subunit. (**A**) The refined complex structures of Stx2B with peptide ligands TF-1. (**B**) The modeled complex structure architectures of Stx2B with its peptide ligands TF-1. (**C**) The non-bonded interaction networks at the Stx2B–TF-1 complex interface.

The interaction between Stx2B and the peptides TF-1 or WA-8 were analyzed by molecular dynamic calculations. The folding modes of the peptides, TF-1 and WA-8, were predicted using the PEP-FOLD server. The peptide ligands were docked to Stx2B with the coarse-grained ZDOCK method, which was further refined by the FlexPepDock approach, resulting in a series of protein–peptide complex conformations. As can be seen in Fig. 6A, the peptide TF-1 structured as a helix motif with its C-terminus inserted into the hole of the Stx2B protein. Conformation sampling revealed that the C-terminus of TF-1 is highly rigid due to the structural constraint from Stx2B, while the N-terminus is far apart from Stx2B body and thus exhibits significant flexibility. A similar interaction mode can also be observed in the Stx2B–WA-8 complex system (S3-A Fig.); although the WA-8 peptide adopts a strand configuration but not a helix structure as in TF-1 to interact with Stx2B.

The Stx2B–TF-1/WA-8 complex conformations with the highest scores were extracted from the conformation sampling and were then addressed with 10-ns molecular dynamics (MD) simulations to achieve equilibrium for the two complexes. Subsequently, the MD-equilibrated systems of Stx2B–TF-1 and Stx2B–WA-8 complexes were immersed into a TIP3P water box and then subjected to an energy minimization procedure of 5000 cycles to obtain the final optimized structures. Both the helical TF-1 peptide (Fig. 6B) and the stranded WA-8 peptide (supplemental Fig. 3B) bound tightly at the entrance of Stx2B hole and were partially inserted into the hole, showing a swinging feature at their rears.

Based on modeled structures, the networks of non-bonded interactions across the complex interfaces of Stx2B with TF-1 (Fig. 6C) and WA-8 (S4-C Fig.) were mapped into 2D page using LigPlot. This Program indicates that the binding of peptide ligands TF-1 and WA-8 to Stx2B is a highly non-selective process, which is primarily derived by hydrophobic effects as only a few specific hydrogen bonds can be found at the complex systems. The amino acid residues (e.g., Asp17, Trp33, Asn34, Gln36, and Pro37) of Stx2B define intensive hydrophobic and van der Waals interaction with the residues Phe2, Trp5, Leu6, Pro7, Phe9, Asn10 and Trp12 of the TF-1 peptide. While the amino acid residues (e.g., Trp33, Asn34, Gln36, Pro37, and Leu38) of Stx2B and the residues Trp1, Trp4, Tyr5, Phe7, Tyr8, and Tyr10 of the WA-8 peptide make up the interaction interface via hydrophobic and van der Waals interactions between the protein and peptide. In addition, several geometrically perfect hydrogen bonds can be observed at the complex interfaces, which are thought to confer additional specificity for the formed complex systems. Hydrogen bonds between residues Asn34 (B) and Asn34 (E) of Stx2B are formed with residues Phe9 and Gln11 of the TF-1 peptide, respectively (Fig. 6C). Residues Asn34 (F) and Ser41 (F) of Stx2B interact via hydrogen bonds with residues Leu12 and Thr8 of the WA-8 peptide, respectively (S4-C Fig.).

## Discussion

In this study, we identified two peptide-based neutralizers (TF-1 and WA-8) that exhibit high affinities for the Stx2B subunit, inhibit Stx2 cytotoxicity, and protect mice or rats from Stx2-caused lethality. However, the peptide, TF-1, had better inhibition activity than WA-8 *in vivo* and *in vitro*. Different Stx2 toxicity models were used to detect the inhibition activity of peptide-based neutralizers *in vivo.* In mice toxicity models, when mice were injected with 5 LD_50_ of Stx2, TF-1 could provide full protection. Even when mice were injected with 10 LD_50_ of Stx2, TF-1 could also provide a 70% protection. In rat toxicity models, TF-1 could completely protect rats from lethal challenge (Fig. 3). TF-1 also displayed good pathological protections on the thymus, kidneys, stomach, and cecum, which have been discussed above (Fig. 4). Hence, the peptide-based neutralizer, TF-1, showed considerable protection even when the administration dose of Stx2 was much higher than the lethal dose. Additionally, watery diarrhea was observed in rat toxicity models but not in mice toxicity models. Thus, rat toxicity models may be a better choice for studies on Stx lethality or effectiveness of inhibitor protection.

Next, the possible mechanism of Stx2-neutralizing action of TF-1 was investigated in this study. First, through the Stx2B-binding assay, we found that TF-1 specifically and directly binds to the B subunit of Stx2. Second, the ^125^I-Stx2/cells binding assay showed that TF-1 efficiently inhibited the cytotoxicity of Stx2 by blocking its Gb3-dependent incorporation into target cells. Then, by injecting ^125^I-Stx2 intravenously into rats with or without TF-1, the effect of TF-1 on the tissue distribution of Stx2 was detected and analyzed. Shorter half-lives, smaller AUC values (Fig. 5A and Table 1), lower percentage of radioactivity precipitation (Fig. 5B, *p* < 0.01), and declined tissue distribution levels (Fig. 5C) were detected in the mice co-injected with TF-1. In conclusion, by the means of TF-1, the toxicokinetics of Stx2 with a medium dose was transformed from a three-compartment model to a two-compartment model just as the toxicokinetics of Stx2 with a low dose^34^. Considering the close relationship between Stx2 concentration in the blood as well as in tissues and its mortality in rats, these results suggest that TF-1 suppressed the lethality of Stx2 by diminishing the deposition of Stx2 in the blood and other tissues and the subsequent fatal damage. It was consistent with the histopathology results that pathological damages of Stx-target tissues (such as the kidney, thymus, adrenal gland, etc.) were minimal in TF-1 co-injected rats. Although the precise molecular mechanism of how TF-1 functions *in vivo* remains to be elucidated, we speculate that TF-1 protected these tissues by interfering with the binding between Stx2 and Gb3 which accelerates the elimination of Stx2.

Finally, to discover the possible action site of peptide-based neutralizers, TF-1 and WA-8, the interaction of the peptide and Stx2B were analyzed by molecular dynamic calculations. Previous reports showed that three distinct receptor-binding (GB3-binding) sites have been identified on the B-pentamer of Stxs, including Stx2^6^,^7^. Site 3 (ASP17, TRP33) involves hydrophobic stacking interactions between Galβ and the indole ring of Trp33 as well as hydrophobic interactions between Galβ and Trp33 of an adjacent monomer^35^. In this study, computer simulations suggested that the majority of TF-1 and WA-8 both bound tightly at the entrance of the Stx2B hole, which is the receptor-binding site 3 of Stx2B. The binding of peptide ligands, TF-1 and WA-8, to Stx2B is primarily derived by hydrophobic effects. In fact, a number of non-polar amino acid residues such as Trp33, Pro37, and Leu38 are found at the entrance region of the Stx2B hole, which define intensive contacts of hydrophobicity and van der Waals interactions with the residues of the TF-1 or WA-8 peptide, forming a large interface between Stx2B and peptide inhibitors. Interesting, the hole of the Stx2B subunit, is also occupied by the C-terminus region of the Stx2A subunit when forming the Stx2A - Stx2B complex. But the binding of the A and B subunits of Stx2 was dynamic process in infected host cells. Thus, the peptides have the change to compete with A subunit to bind to the B subunit, which may be one of action mechanism of the peptide inbibitors.

To elucidate the mechanism by which TF-1 binds to 2BH, the binding between TF-1 or WA-8 and a series of 2BH mutants with mutations in the receptor binding region was examined using the ELISA assay. Under the same conditions, the binding of TF-1 to 2BH-D17E, W33A or N34A, all of which are site 3 mutant, were markedly reduced to 32%, 69% or 41% of that of 2BH (S5-A Fig.), whereas the levels of binding of TF-1 to 2BH-D16E or 2BH-G61A, site 1 or site 2 mutant, were no significance difference with the wild type 2BH. Furthermore, the binding of WA-8 to 2BH wild type or mutant were similar to the binding of TF-1 to 2BH (S5-B Fig.). These results indicate the main contribution of sites 3, but not site 1 or site 2, to the binding of TF-1 or WA-8 to the Stx2 B subunit. Interestingly, recent studies have shown that site 3 plays a more important role in high-affinity binding to the Gb3 receptor on the cell surface and intracellular localization compared to site 1 (Asp16 and Trp29) and site 2 (Ser54 and Gly61)^7^,^20^,^23^. In this study, TF-1 or WA-8 was found to bind to the Stx2B subunit exclusively through site 3, possibly contributing to the potent inhibitory effect against Stx2 cytotoxicity and lethality. Thus, site 3 of Stx could be an efficient target for development of Stx neutralizers that function *in vivo*.

In summary, we identified a Stx neutralizer, TF-1, that was active *in vitro* and *in vivo*. It bound to Stx2 and inhibited its Gb3-dependent incorporation into target cells *in vitro*. Furthermore, TF-1 protected mice or rats from challenge with a fatal dose of Stx2 by diminishing the deposition of Stx2 in the blood and other tissues. Although the precise mechanism of TF-1 inhibition of Stx2 lethality *in vivo* remains to be elucidated, the peptide-based neutralizer TF-1 provides a new strategy to eliminate Stx2 from the body.

## Additional Information

**How to cite this article**: Li, T. *et al.* A potential therapeutic peptide-based neutralizer that potently inhibits Shiga toxin 2 *in vitro* and *in vivo*. *Sci. Rep.* **6**, 21837; doi: 10.1038/srep21837 (2016).

## Supplementary Material

Supplementary Figures S1-S5

## Figures and Tables

**Table 1:** Peptides binding to 2BH after the fourth round of selection from the phage display library.

Names	Sequence	Molecular weight	Theoretical pI
TF-1	TFNMWLPTFNQW	1625.8	5.19
TL-2	TLPSFFSTWWPS	1496.6	5.19
WF-3	WFHIPFLYVPAS	1475.8	6.74
TW-4	TWFDFYTWAPRL	1643.8	5.50
FT-5	FTPYTHLSYPFL	1526.7	6.74
TY-6	TYPWPWHFPYS	1521.7	6.4
NF-7	NFFAPWMWLPS	1573.9	5.52
WA-8	WAPWYSFTSYHL	1598.7	6.74
KF-9	KFTHHNVPPNWF	1523.7	8.76
HS-10	HSILYTNLASHR	1411.6	8.76
HN-11	HNPMSFPTPRLY	1459.7	8.75
WT-12	WTPLHWINTTPR	1521.8	9.76
QT-13	QTLASWPWSFRY	1582.7	8.75
IM-14	IMTKLTVNLNHA	1353.7	8.76
RR-15	RRSPGWIHSYGN	1429.6	10.84

**Table 2:** Toxicokinetic parameters obtained with 25.5 μg/kg intravenous ^125^I-Stx2 or ^125^I-Stx2 mixed with TF-1 in Wistar rats^a^.

Parameters	Dose	
	Stx2 (25.5 μg/kg)	Stx2 (25.5 μg/kg) + TF-1 (5 mg/kg)
t1/2α (h)	0.098	0.094
t1/2β (h)	1.204	1.684** c
t1/2γ (h)	27.876	−
V1 (l/kg)	0.06	0.114** b
CL (l/h/kg)	0.024	0.082** b
AUC(0-t) (mg/l·h)	762.077	232.057** b
AUC(0-∞) (mg/l·h)	846.395	243.545** b
Compartment model	three	two

^a^Plasma Stx2 levels (Fig. 5A) were used to calculate toxicokinetic parameters with the DAS 2.0 software.

^b^*p* < 0.01, compared with 25.5 μg/kg dose Stx2 group.

^c^*p* < 0.01, t_1/2_β of intervention group compared with t_1/2_γ of the challenged group receiving a 25.5 μg/kg dose of Stx2.
