# Supplementary material for: A potential therapeutic peptide-based neutralizer that potently inhibits Shiga toxin 2 in vitro and in vivo
Source: Sci Rep. 2016 Feb 23;6:21837. doi: 10.1038/srep21837 (PMC4763182; doi:10.1038/srep21837)

A potential therapeutic peptide-based neutralizer that potently inhibits Shiga toxin 2 *in vitro* and *in vivo*

Tao Li^1¶^, Wei Tu^1¶^, Yuenan Liu^1^, Peng Zhou^4,5^, Kun Cai^1^, Zhan Li^1^, Xiong Liu^1^, Nianzhi Ning^1^, Jie Huang^1^, Shenghan Wang^6^, Jian Huang^4,5^, Hui Wang^1,2,3^ *

S1 Fig. Quality control of synthesized TF-1 and WA-8. A: mass spectrometry analysis of TF-1. B: high performance liquid chromatography analysis of TF-1. C: mass spectrometry analysis of WA-8. D: high performance liquid chromatography analysis of WA-8.


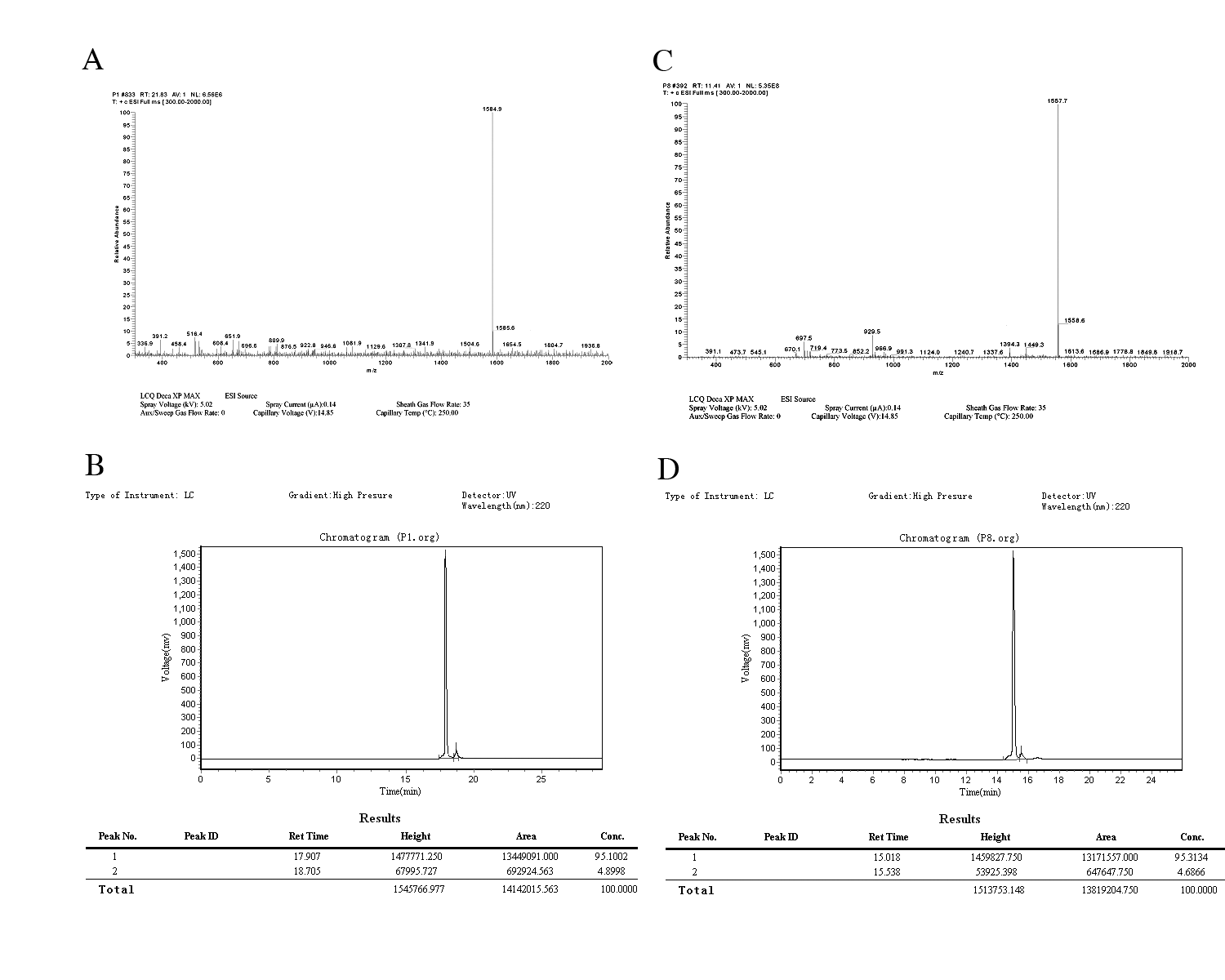


S2 Fig. The morphological and anatomical studies of rats with or without TF-1 treatment. Condition of diarrhea in rats (A1-A3), condition of stomach and cecum in rats (B1-B3), condition of kidney in rats (C1-C3).


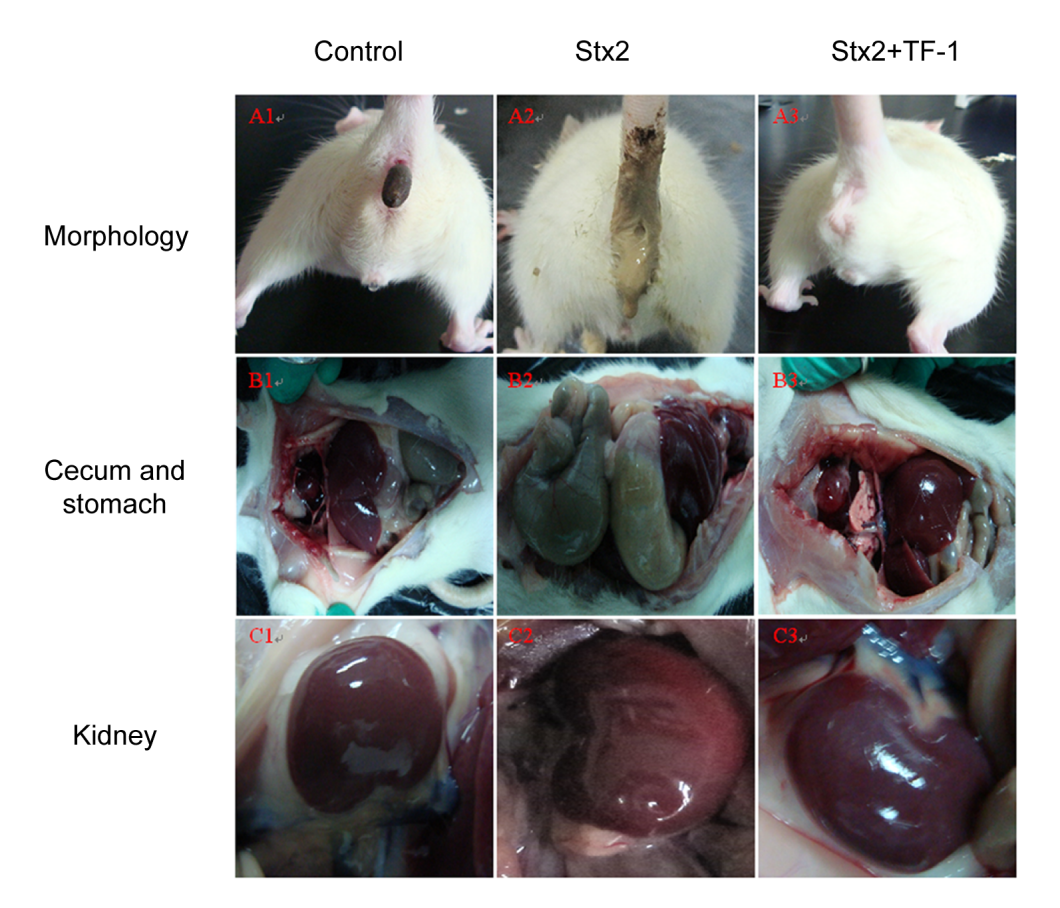


S3 Fig. Histopathological studies of rats in the cerebellum, cerebrum, liver, spleen, heart, intestine, stomach, fat, spinal cord, and testis.


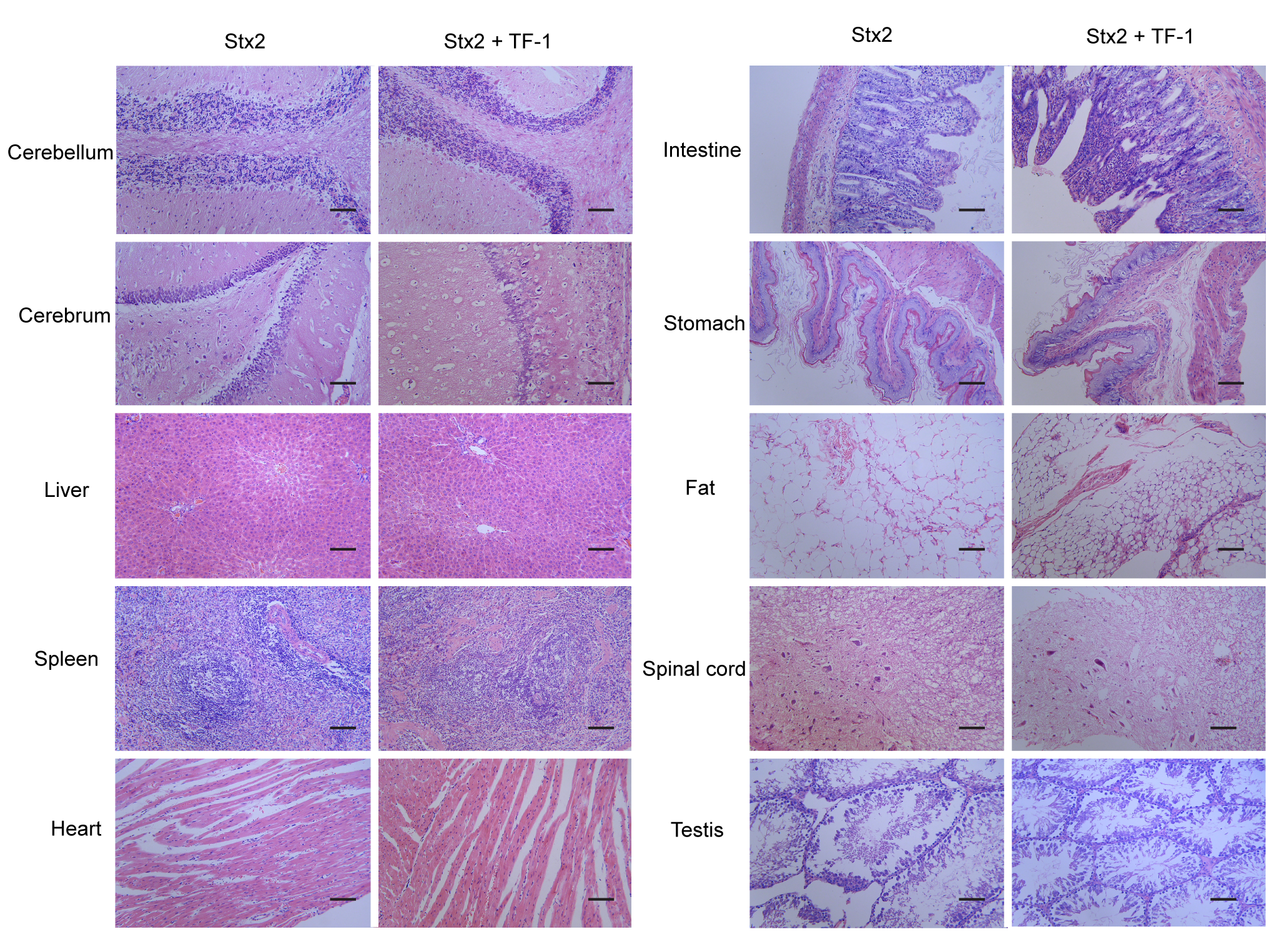


S4 Fig. Computer-simulated binding mode between WA-8 and Stx2B subunit: (A) The refined complex structures of Stx2B with peptide ligands WA-8. (B) The modeled complex structure architectures of Stx2B with its peptide ligands WA-8. (C) The non-bonded interaction networks at Stx2B-WA-8 complex interface.


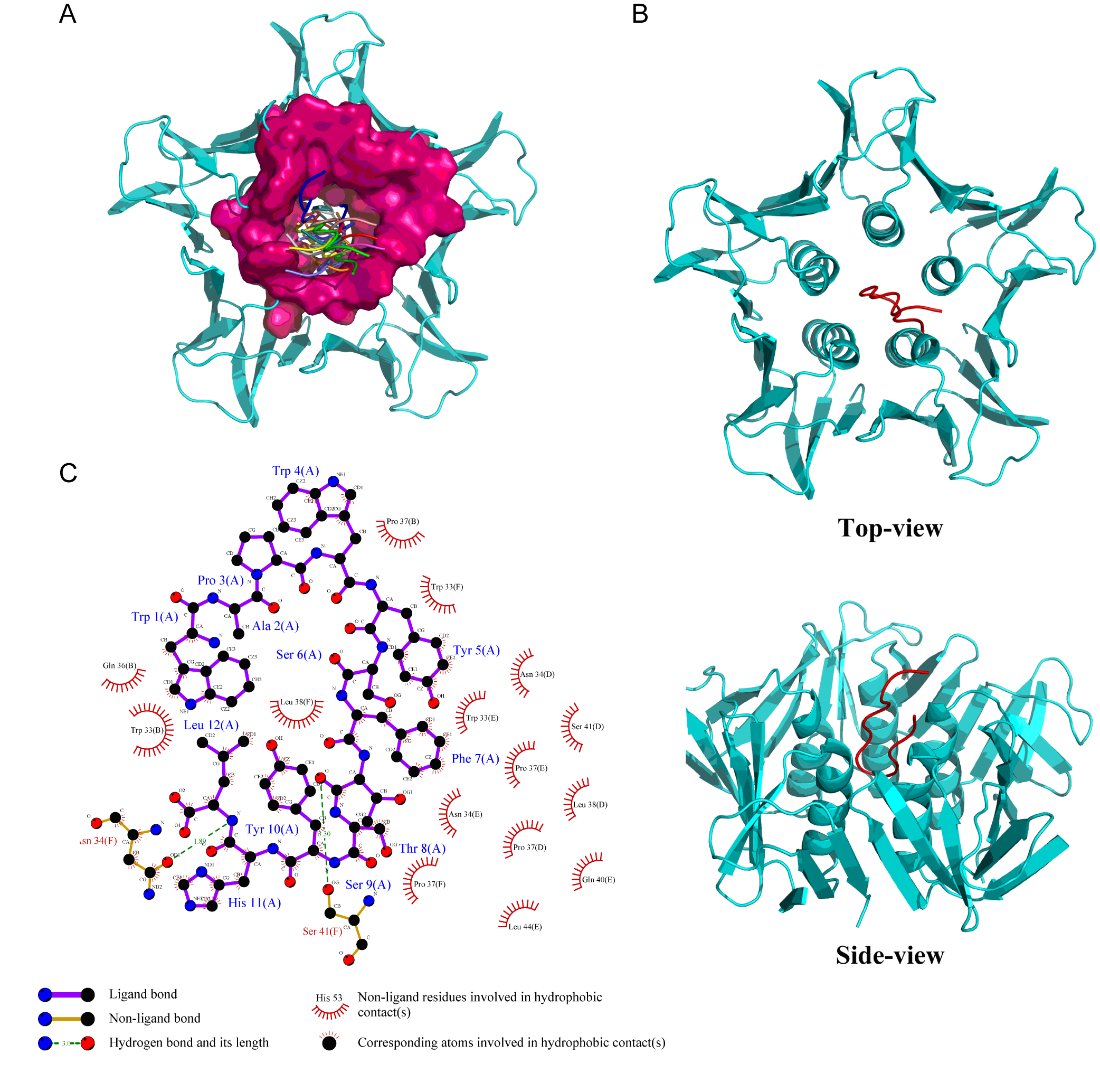


S5 Fig. The binding of TF-1 (A) or WA-8 (B) with Stx2B or Stx2B mutant were examined using ELISA (mean ± standard error, n = 3; ** P < 0.01, Tukey’s test). D16E, D17E, W33A, N34A and G61A, Stx2B with a substitution of Asp, Asp, Trp, Asn, Gly to Glu or Ala at positions 16, 17, 33, 34, and 61, respectively. OD490, optical density at 490 nm.


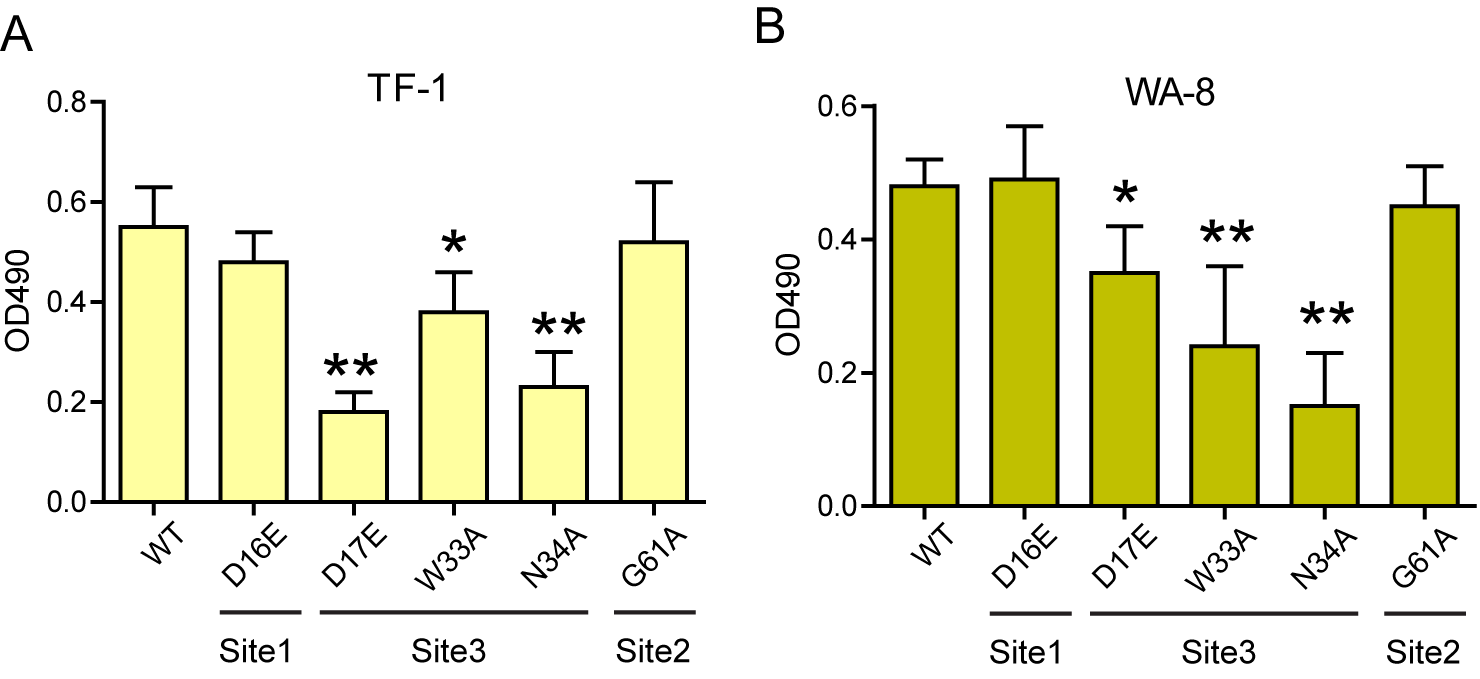

Supplement: Supplementary Figures S1-S5 [file srep21837-s1.doc]
